# Global burden of type 2 diabetes attributable to SARS-CoV-2 infection

**DOI:** 10.3389/fimmu.2026.1969341

**Published:** 2026-10-16

**Authors:** Jingjing Li, Guangming Liu, Jianzhou Yang, Haimiao Ma, Xiaoyan Wang, Ziying Jiang, Yuanhao Liang, Xiaofeng He

**Affiliations:** 1 Institute of Evidence-Based Medicine, Heping Hospital Affiliated to Changzhi Medical College, Changzhi, China; 2 Department of Clinical Laboratory, Heping Hospital Affiliated to Changzhi Medical College, Changzhi, China; 3 Department of Public Health and Preventive Medicine, Changzhi Medical College, Changzhi, China; 4 Department of Medical Statistics, School of Public Health, Zhejiang Chinese Medical University, Hangzhou, China; 5 School of Public Health, Southern Medical University, Guangzhou, China

**Keywords:** COVID-19, global burden of disease, population attributable fraction, SARS-CoV-2, type 1 diabetes, type 2 diabetes

## Abstract

**Objective:**

We aimed to quantify the association between severe acute respiratory syndrome coronavirus 2 (SARS-CoV-2) infection and the risk of incident diabetes. We also aimed to estimate the global burden of incident type 2 diabetes (T2D) attributable to SARS-CoV-2 infection.

**Methods:**

We conducted a systematic review and meta-analysis of cohort studies identified through PubMed, Embase, Web of Science and CENTRAL from inception to 29 January 2026. Adjusted relative risks (RRs) for incident T2D and type 1 diabetes (T1D) following SARS-CoV-2 infection were pooled using random-effects models. Population attributable fractions (PAFs) and attributable T2D cases were estimated by integrating pooled risk estimates with Global Burden of Disease (GBD) 2023 data on COVID-19 prevalence and T2D incidence.

**Results:**

Fifteen cohort studies involving approximately 48.9 million participants were included. SARS-CoV-2 infection was associated with an increased risk of incident T2D (13 studies; pooled RR, 1.25 [95% CI, 1.16–1.34]), although substantial heterogeneity was observed and the 95% prediction interval (PI) included the null (0.94–1.66). In subgroup analysis, the association was confined to mixed-variant studies (RR, 1.29 [95% CI, 1.23–1.36]; 95% PI, 1.09–1.54), whereas studies evaluating a single variant (Delta or Omicron) showed no excess risk (RR, 1.00 [95% CI, 0.97–1.03]). The association with incident T1D did not reach conventional statistical significance (10 studies; pooled RR, 1.13 [95% CI, 0.99–1.30]). Globally, 24.53 million incident T2D cases occurred in 2023. The PAF attributable to COVID-19 of 1.12% (95% CI 0.90, 1.35), corresponding to 274,889 (95% CI 220,360, 331,698) incident T2D cases attributable to SARS-CoV-2 infection. The largest attributable burdens were observed in India, the USA, and China.

**Conclusions:**

SARS-CoV-2 infection was associated with a modest increase in pooled T2D risk. This association was primarily observed in mixed-variant studies, for which the 95% PI excluded the null. However, the overall pooled estimated was accompanied by substantial between-study heterogeneity, and its 95% PI crossing the null, indicating uncertainty in the association across populations and settings. At the population level, this excess risk translated into an estimated 275,000 incident T2D cases attributable to COVID-19 worldwide in 2023, highlighting the need for ongoing metabolic surveillance following infection.

**Systematic Review Registration:**

https://www.crd.york.ac.uk/PROSPERO/view/CRD420261307969, identifier CRD420261307969.

## 1 Introduction

Coronavirus disease 2019 (COVID-19), caused by the novel severe acute respiratory syndrome coronavirus 2 (SARS-CoV-2), has evolved into one of the most consequential global health crises of the twenty-first century (1). Since its emergence in late 2019, the pandemic has resulted in substantial morbidity and mortality worldwide and has placed an unprecedented burden on health systems and societies. As of January, 2026, more than 775 million confirmed cases and over 7 million deaths have been reported globally (2). Beyond its acute clinical manifestations, increasing attention has been directed toward the long-term health consequences of SARS-CoV-2 infection (3). A considerable proportion of individuals experience persistent or newly emerging symptoms following recovery from the acute phase of infection, a condition commonly referred to as post-COVID-19 condition or long COVID (4, 5). These sequelae affect multiple organ systems and include cardiovascular, neurological, thrombotic, and metabolic complications (6).

Diabetes mellitus is among the most prevalent and burdensome chronic diseases globally, affecting hundreds of millions of individuals and contributing substantially to premature mortality, disability, and health-care costs (7, 8). The interaction between COVID-19 and diabetes appears to be complex (9). Individuals with pre-existing diabetes have consistently been shown to experience a higher risk of severe COVID-19 outcomes, including hospitalization, intensive care unit admission, and mortality (10–15). However, recent cohort evidence showing no statistically significant association between diabetes and risk of SARS-CoV-2 infection after adjustment for relevant factors (16). Conversely, growing evidence suggests that SARS-CoV-2 infection may increase the risk of newly diagnosed diabetes (17, 18). Observational studies conducted in diverse populations have reported elevated rates of incident diabetes following COVID-19 infection compared with non-infected individuals. Both type 1 and type 2 diabetes have been implicated, although the underlying mechanisms remain incompletely understood (19, 20). Proposed biological pathways include direct viral tropism for pancreatic β-cells, immune-mediated β-cell injury, systemic inflammation, glucocorticoid exposure during treatment, and persistent metabolic dysregulation associated with post-COVID-19 condition (21, 22). Furthermore, the epidemiological impact of SARS-CoV-2 infection has evolved with vaccination, population immunity, viral variants, and clinical management, potentially altering the risk of post-acute diabetes (23, 24). Thus, diabetes risk may vary by pandemic period, vaccination status, viral lineage, and population immunity.

Given the enormous scale of the COVID-19 pandemic, even a modest increase in diabetes risk associated with SARS-CoV-2 infection could translate into a substantial additional burden of diabetes at the population level. However, the magnitude of this burden has not yet been comprehensively quantified. Population attributable fraction (PAF) provides an epidemiological framework to estimate the proportion of disease cases that can be attributed to a specific exposure within a population, assuming a causal relationship (25). When combined with reliable estimates of exposure prevalence and relative risk, PAF allows the estimation of the number of incident cases attributable to that exposure (26). Although several systematic reviews and meta-analyses have examined the association between SARS-CoV-2 infection and incident diabetes (27–29), the rapidly expanding body of evidence warrants an updated synthesis to generate robust risk estimates suitable for population-level burden assessment.

In this study, we conducted a comprehensive systematic review and meta-analysis to quantify the association between SARS-CoV-2 infection and the risk of newly diagnosed diabetes. In parallel, we analyzed data from the Global Burden of Disease (GBD) 2023 to characterize the global, regional, and national burden of type 2 diabetes (T2D), including incidence and disability-adjusted life years (DALYs), in 2023 (30). By integrating pooled relative risk estimates from the meta-analysis with global epidemiological estimates of SARS-CoV-2 infection prevalence and diabetes burden, we further quantified the population attributable fraction and the number of incident T2D cases attributable to COVID-19 at global and regional levels. By estimating the long-term diabetes burden associated with SARS-CoV-2 infection, this study provides evidence to support post-pandemic public health strategies, diabetes prevention initiatives, and health-system planning.

## 2 Research design and methods

### 2.1 Study design and reporting standards

This systematic review and meta-analysis were conducted in accordance with the Preferred Reporting Items for Systematic Reviews and Meta-Analyses (PRISMA) guidelines (31) and the Meta-analysis of Observational Studies in Epidemiology (MOOSE) reporting recommendations (32) (**Appendix Tables S1**, **S2**). The study protocol was prospectively registered in the PROSPERO database on Feb 13, 2026 (registration number: CRD420261307969).

### 2.2 Search strategy and eligibility criteria

A comprehensive literature search was performed in PubMed, Embase, the Cochrane Central Register of Controlled Trials (CENTRAL), and Web of Science from database inception to January 29, 2026. The search strategy incorporated terms related to diabetes mellitus, COVID-19, SARS-CoV-2 infection, and cohort study designs. No restrictions were applied regarding publication year or language. The complete search strategies for all databases are provided in **Appendix Table S3**.

We included observational cohort studies that evaluated the association between SARS-CoV-2 infection and the risk of newly diagnosed diabetes compared with non-COVID-19 populations. Eligible studies were required to report adjusted effect estimates, including relative risks (RRs) or hazard ratios (HRs), for incident T1D or T2D. To minimize potential misclassification due to transient metabolic disturbances during acute COVID-19 illness, studies were required to assess diabetes occurrence at least 28 days after SARS-CoV-2 diagnosis. Detailed eligibility criteria used for title and abstract screening are provided in **Appendix Table S4**.

### 2.3 Data extraction and quality assessment

Two investigators (JY and JL) independently extracted data and assessed study quality. Extracted information included study characteristics, participant demographics, exposure definition, diabetes outcome definition, follow-up duration, effect estimates, and covariates included in adjusted models. Any discrepancies were resolved through discussion with a third investigator (XH). Study authors were contacted when additional information or clarification was required.

The methodological quality of included studies was evaluated using the Newcastle–Ottawa Scale (NOS) (33). The risk of bias was categorized as low (7–9 stars), moderate (4–6 stars), or high (1–3 stars), consistent with previously established criteria.

### 2.4 Estimates of pooled relative risks

Quantitative synthesis was performed using Stata version 12.0 (StataCorp, College Station, TX, USA). Adjusted effect estimates were pooled using a random-effects model, which accounts for between-study variability by assuming that the underlying effect size may differ across studies due to variations in populations, study designs, exposure definitions, and healthcare settings (34). Summary estimates were presented as pooled adjusted RRs with corresponding 95% confidence intervals (CIs). When studies reported HRs, these estimates were considered comparable to RRs under the assumption of relatively low absolute incidence of newly diagnosed diabetes during follow-up. Statistical heterogeneity was assessed using Cochran’s Q statistic and the I² statistic, with substantial heterogeneity defined as *P* < 0.01 and/or *I²* >50%. Prediction intervals (PIs) were additionally calculated to estimate the expected range of true effects in future studies.

Sensitivity analyses were conducted to assess the robustness of the pooled estimates. First, a leave-one-out analysis was performed by sequentially excluding each individual study. Second, predefined sensitivity analyses were conducted to evaluate the influence of clinically distinct subgroups. For T2D, the unvaccinated cohort from Taylor et al. was excluded because of its distinct lack of vaccine-induced immunity. For T1D, Ward et al. and the unvaccinated cohort from Taylor et al. were simultaneously excluded. Robustness was evaluated according to whether the direction and interpretation of the prediction intervals remained consistent after exclusion of these subgroups.

Potential publication bias was assessed using Begg’s funnel plot (35) and Egger’s regression test (36). The certainty of evidence for the association between SARS-CoV-2 infection and incident diabetes was evaluated using the Grading of Recommendations Assessment, Development, and Evaluation (GRADE) framework (37). A two-sided *P* value<0.05 was considered statistically significant.

We conducted exploratory univariable meta-regression to identify sources of heterogeneity in the T2D analysis. Covariates included viral lineage (mixed variants vs. Delta or Omicron variant only), SARS-Cov-2 exposure ascertainment (PCR-confirmed vs. electronic health record/diagnostic code-based), and follow-up duration. Because only 13 studies were available and these covariates were potentially correlated, multivariable meta-regression would be underpowered and prone to overfitting and multicollinearity; therefore, only univariable models were fitted.

### 2.5 Global burden of disease data sources

Data on the incidence and disability-adjusted life years (DALYs) of T2D in 2023 were obtained from the GBD 2023 study through the Global Health Data Exchange (GHDx) (30). Estimates were extracted at global, regional, and national levels. The GBD study applies a standardized analytical framework that integrates data from multiple sources, including population-based surveys, disease registries, administrative health databases, vital registration systems, and published scientific literature. These data are processed using advanced statistical modeling approaches to generate comparable estimates across locations and over time. T2D was defined according to the GBD cause hierarchy and corresponding International Classification of Diseases (ICD) coding framework (30). Age-standardized incidence rates (ASIRs) and age-standardized DALY rates (ASDRs) were reported per 100,000 population and calculated using the GBD standard population to enable comparisons across populations with different age structures.

To evaluate the relationship between socioeconomic development and the burden of T2D, we incorporated the Socio-demographic Index (SDI), a composite indicator reflecting national socioeconomic development (30). SDI is calculated as the geometric mean of three equally weighted components: lag-distributed income per capita, mean years of schooling among individuals aged ≥15 years, and total fertility rate among women younger than 25 years. Based on SDI values, 204 countries and territories were categorized into five SDI quintiles: low, low-middle, middle, high-middle, and high SDI (**Appendix Table S5**).

Associations between SDI and T2D burden indicators (ASIR and ASDR) were assessed using Spearman’s rank correlation analysis at both regional and national levels. Spearman’s correlation coefficient (ρ) and corresponding *P* values were calculated to evaluate monotonic relationships between socioeconomic development and T2D burden. This non-parametric approach was selected because it does not require assumptions of data normality and is appropriate for evaluating potential non-linear associations involving continuous or ordinal variables.

### 2.6 Estimation of population attributable fraction and diabetes incidence attributable to COVID-19 infection

To quantify the population-level burden of diabetes attributable to COVID-19 infection, we estimated the population attributable fraction (PAF) and the corresponding number of incident diabetes cases attributable to COVID-19 infection. PAF represents the proportion of disease cases in a population that can be attributed to a specific exposure, assuming a causal relationship between the exposure and the outcome. The PAF was calculated using the standard epidemiological formula (38):


$$Population\;attributable\;fraction\left(PAF\right)=\frac{P\left(RR-1\right)}{1+P\left(RR-1\right)}$$

where *P* denotes the prevalence of COVID-19 infection and RR represents the represents the pooled relative risk of incident diabetes associated with COVID-19 infection obtained from the meta-analysis. The prevalence of COVID-19 infection and diabetes incidence was obtained from Global Burden of Disease Study 2023 (39). The estimated PAF was applied to the total number of incident diabetes cases to estimate the number of diabetes cases attributable to COVID-19 infection. The number of attributable cases was calculated as:


COVID−19 attributable diabetes incidence=Total diabetes incidence×PAF

Stratified analyses were further conducted by sex and age groups to assess variation in the attributable burden across different populations. To account for uncertainty in the pooled relative risks, 95% uncertainty intervals for the attributable burden were estimated using Monte Carlo simulations by sampling from the distribution of the pooled risk estimates. All statistical analyses and mapping were performed using R software, version 4.3.0 (R Foundation for Statistical Computing), with statistical significance defined as *P* < 0.05.

### 2.7 Role of funding source

The funder of this study had no role in study design, data collection, data analysis, data interpretation, or writing of the report. All authors had full access to all the data in the study and accepted responsibility to submit for publication.

### 2.8 Ethics statement

Ethical approval was not required for this study because it is a systematic review and meta-analysis based exclusively on previously published data. All included studies were conducted in accordance with relevant ethical standards and had obtained appropriate institutional ethics approval and informed consent from participants, as reported in the original publications. No individual-level identifiable data were used in this study.

## 3 Results

### 3.1 Study selection and characteristics

The literature search identified 23,868 records, of which 6,921 duplicates were removed (**Figure 1**). After title and abstract screening, 67 articles underwent full-text review. Fifty-two articles were excluded for reasons detailed in **Appendix Table S6**, leaving 15 eligible articles (**Appendix Table S7**) (24, 40–53). These included 13 studies (48,437,729 participants) evaluating incident T2D and 10 studies (48,924,267 participants) evaluating incident T1D following SARS-CoV-2 infection (**Appendix Table S8**). All included studies were assessed as low risk of bias according to the Newcastle–Ottawa Scale (**Appendix Table S9**). Details of covariate adjustment are provided in **Appendix Table S10**.

**Figure 1:**
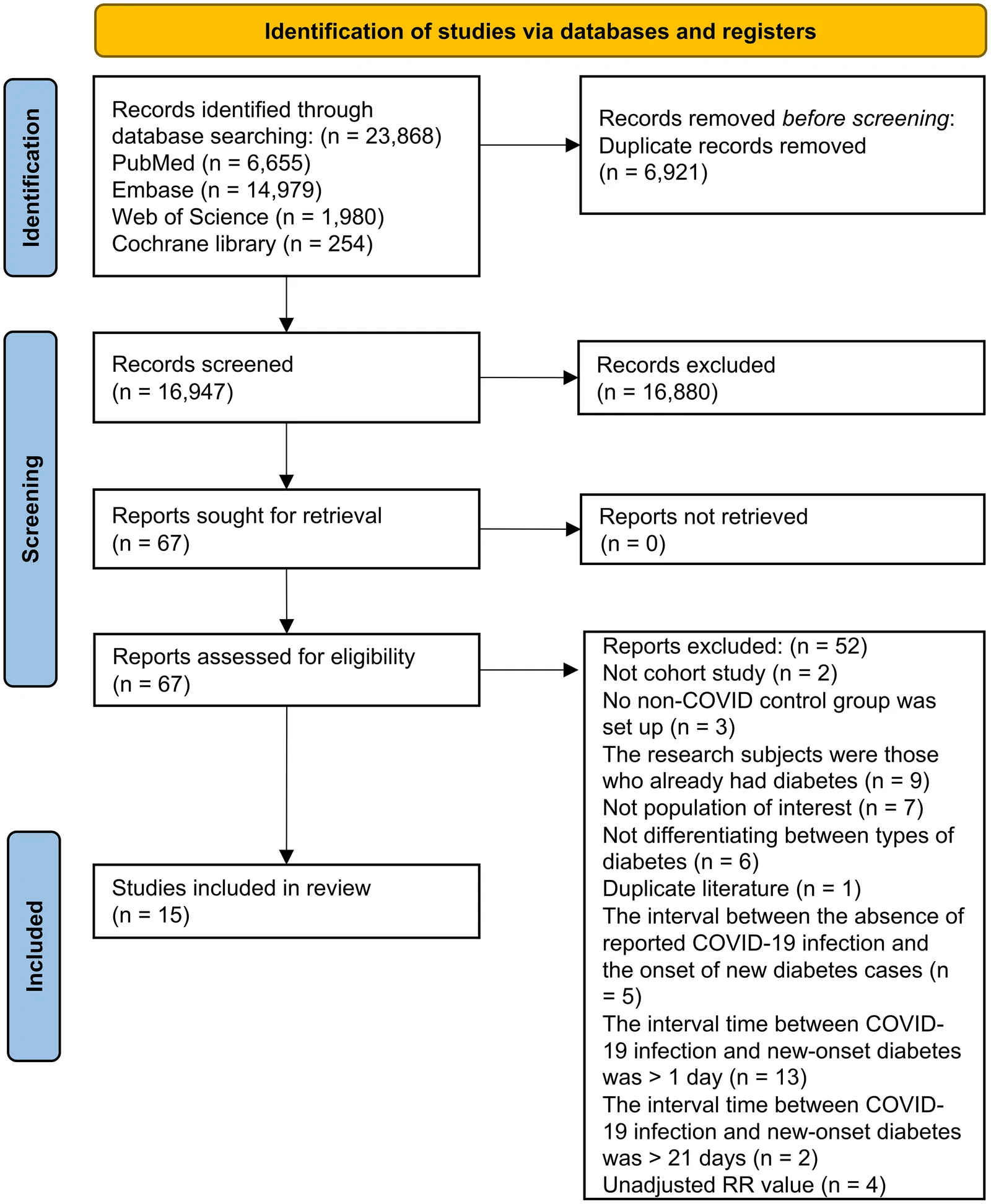
Study selection process for the systematic review and meta-analysis.

### 3.2 Association between SARS-CoV-2 infection and T2D risk

Across 13 studies, SARS-CoV-2 infection was associated with a significantly increased risk of incident T2D (RR 1.25, 95% CI 1.16–1.34; *I²* = 96.8%; **Figure 2**). However, the 95% prediction interval (PI) included the null value (0.94–1.66). In exploratory univariable meta-regression, viral lineage (*P* = 0.003) was a significant source of between-study heterogeneity, whereas exposure ascertainment (*P* = 0.850) and follow-up duration (*P* = 0.505) were not. In prespecified subgroup analysis by viral lineage, studies evaluating a single variant (Delta or Omicron) showed no association with incident T2D (RR 1.00, 95% CI 0.97–1.03; *I²* = 0.0%), whereas mixed-variant studies reported a significantly elevated risk (RR 1.29, 95% CI 1.23–1.36), with a 95% PI of 1.09–1.54 (**Figure 2**). The between-subgroup difference was statistically significant (*P* < 0.001). Leave-one-out analyses demonstrated that no individual study materially influenced the pooled estimate (**Appendix Figure S1**). Exclusion of the extreme unvaccinated cohort reported by Taylor et al. yielded similar results (RR 1.28, 95% CI 1.22–1.34; 95% PI 1.08–1.50; **Appendix Figure S2**). No evidence of publication bias was detected within the mixed-variant subgroup (Egger’s test, *P* = 0.761; **Appendix Figure S3**). According to the GRADE framework, the certainty of evidence was rated as moderate (**Appendix Table S11**).

**Figure 2:**
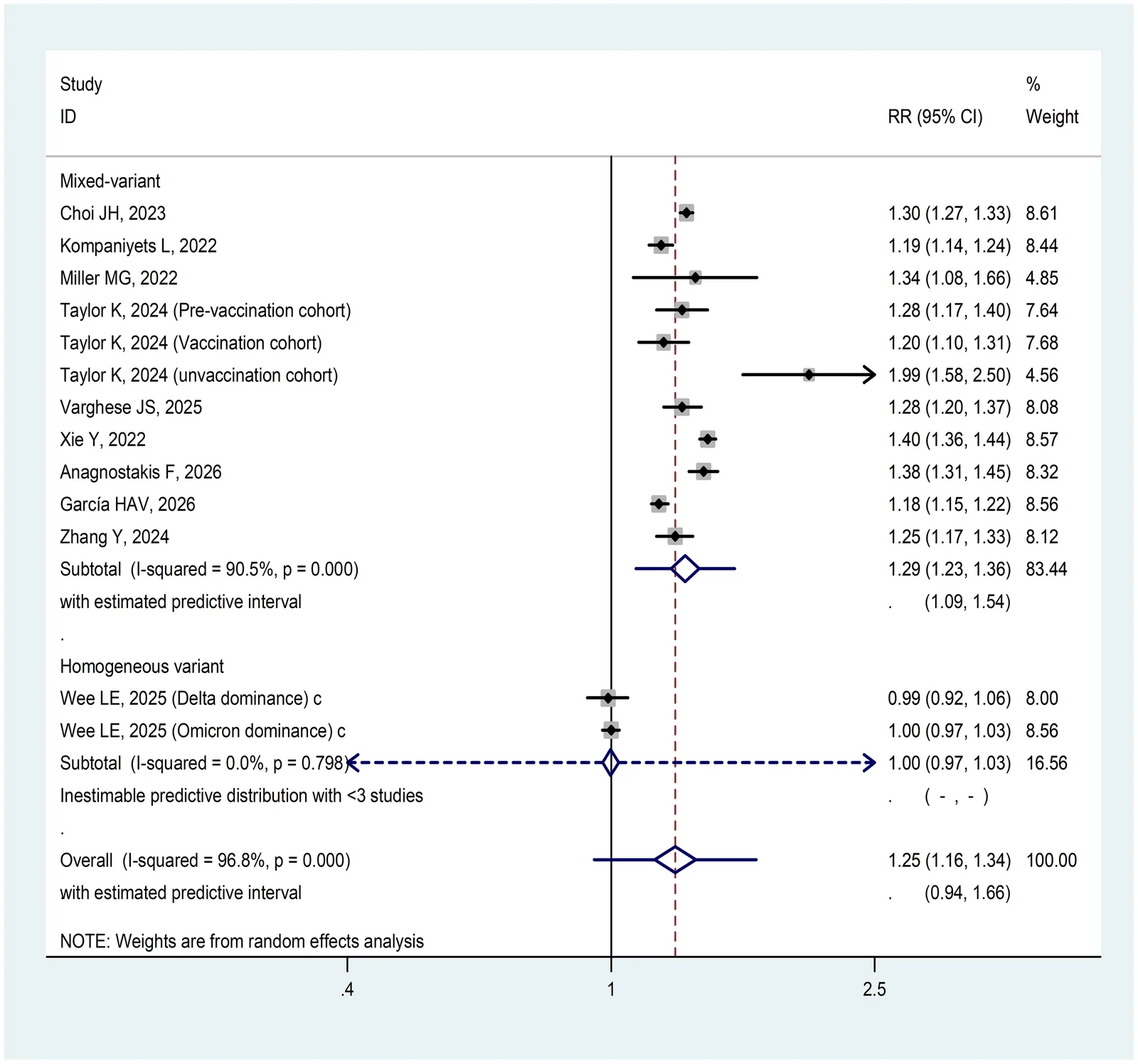
Forest plot of pooled relative risks for incident type 2 diabetes associated with SARS-CoV-2 infection.

### 3.3 Association between SARS-CoV-2 infection and T1D risk

The pooled analysis of 10 studies suggested a modestly increased risk of incident T1D following SARS-CoV-2 infection, although the association did not reach conventional statistical significance (RR 1.13, 95% CI 0.99–1.30; *I²* = 83.7%; **Figure 3**). Thus, the point estimate corresponded to a 13% relative increase in T1D risk, but the confidence interval included the null. The corresponding 95% prediction interval (0.72–1.77) further indicated substantial uncertainty regarding the magnitude and direction of the association across different populations (**Figure 3**). Additionally, subgroup findings suggested that the association was more apparent in studies conducted before or without widespread vaccination, whereas it was attenuated in vaccinated populations. Age-stratified subgroup analyses showed no significant associations across any age category, and no significant between-subgroup differences were observed (*P* > 0.05; **Figure 3**). Sensitivity analyses confirmed the robustness of the findings. Neither leave-one-out analyses (**Appendix Figure S4**) nor simultaneous exclusion of two influential studies (Ward et al. and the unvaccinated cohort from Taylor et al.) materially altered the pooled estimate (**Appendix Figure S5**). No evidence of publication bias was identified (Egger’s test, *P* = 0.250; **Appendix Figure S6**). The certainty of evidence for the T1D analysis was rated as moderate according to the GRADE framework (**Appendix Table S10**).

**Figure 3:**
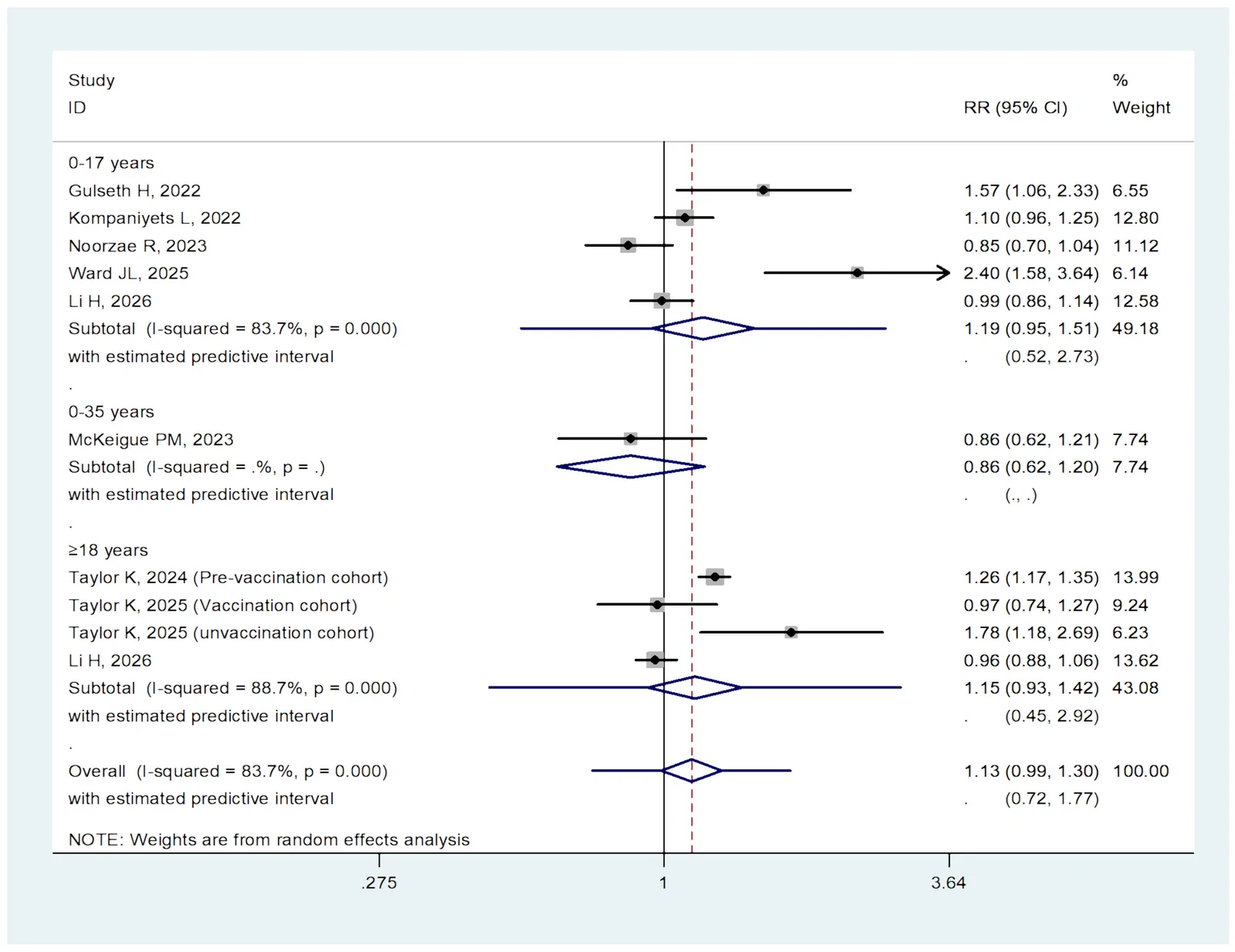
Association of SARS-CoV-2 infection with type 1 diabetes, 2022 to 2026.

### 3.4 Global burden of T2D in 2023

In 2023, an estimated 24.53 million (95% UI, 22.77–26.33 million) incident cases of T2D occurred globally, corresponding to an age-standardized incidence rate (ASIR) of 281.2 (95% UI, 260.0–302.0) per 100,000 population (**Appendix Table S12**). The global burden reached 87.04 million (95% UI, 72.37–103.89 million) DALYs, with an age-standardized DALY rate (ASDR) of 956.7 (95% UI, 794.4–1142.4) per 100,000 population (**Appendix Table S12**). Disease burden varied substantially across SDI regions. Low-middle SDI regions had the highest ASIR (339.3 per 100,000) and ASDR (1624.3 per 100,000), whereas high-SDI regions had the lowest ASDR (642.8 per 100,000). At the regional level, the highest incidence rates were observed in North Africa and the Middle East, Oceania, and Central Latin America, while the greatest DALY burdens occurred in Oceania, Southern Sub-Saharan Africa, and Central Latin America (**Appendix Table S12**). Nationally, India, China, and the United States accounted for the largest numbers of incident cases and DALYs (**Appendix Table S13**). Geographic maps revealed marked international variation in both incidence and DALY burden (**Figures 4A, B**). Associations between disease burden and SDI demonstrated considerable heterogeneity across levels of socioeconomic development (**Figures 4C, D**).

**Figure 4:**
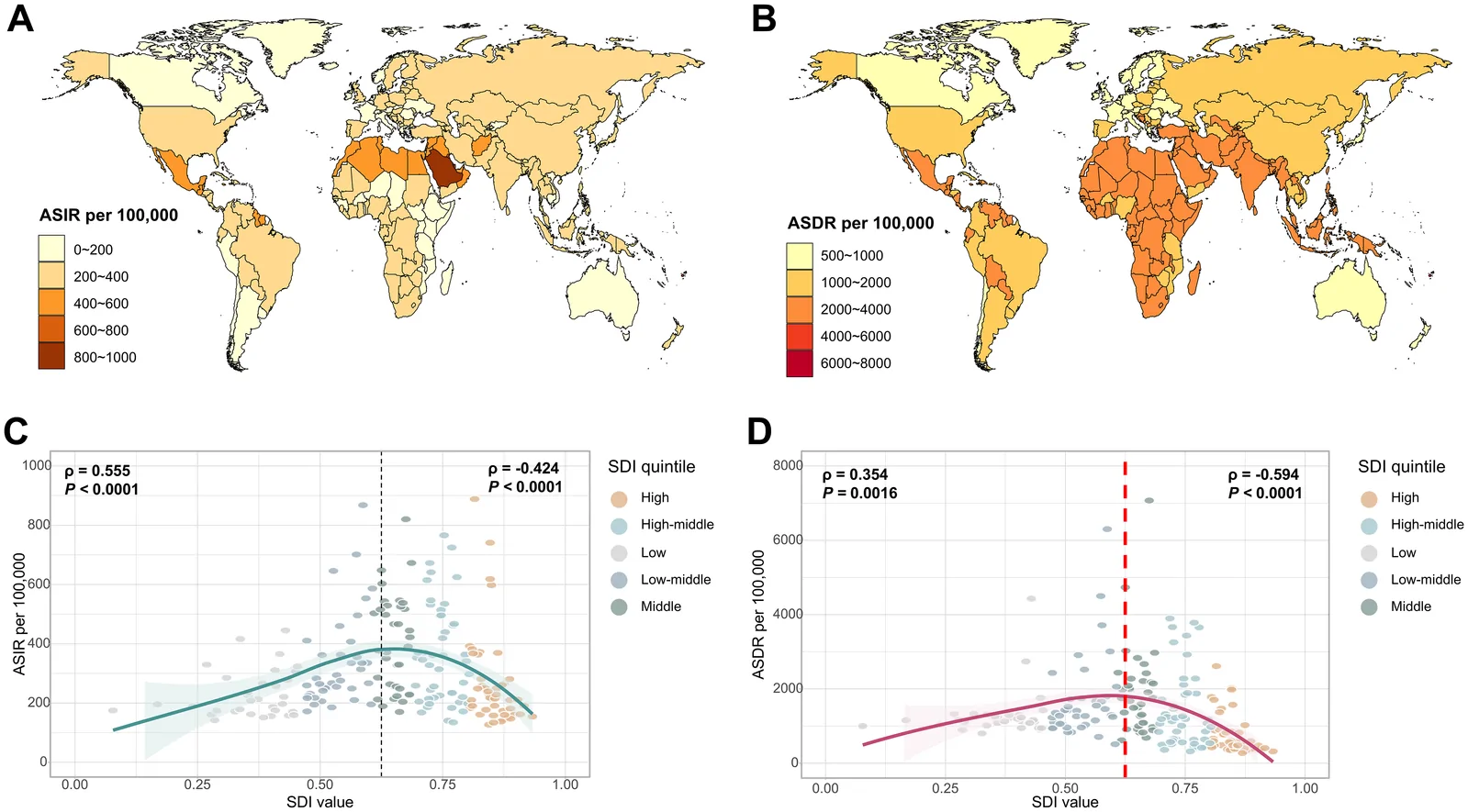
Global distribution of age-standardized incidence and DALY rates of type 2 diabetes and their associations with socio-demographic development in 2023. **(A)** Age-standardized incidence rate (ASIR) of type 2 diabetes per 100,000 population across 204 countries and territories. **(B)** Age-standardized DALY rate (ASDR) per 100,000 population. **(C)** Association between national ASIRs and socio-demographic index (SDI). **(D)** Association between national ASDRs and SDI. ASDR, age-standardized disability-adjusted life year rate; ASIR, age-standardized incidence rate; DALY, disability-adjusted life year; GBD, Global Burden of Disease; SDI, socio-demographic index.

### 3.5 Global burden of T2D attributable to COVID-19 infection

In 2023, an estimated 274,889 incident T2D cases (95% CI: 220,360–331,698) were attributable to COVID-19 infection worldwide, arising from approximately 312.3 million infections in a global population of 8.07 billion. The global prevalence of COVID-19 infection was 3.87% (95% CI: 2.41–6.34%), corresponding to a PAF of 1.12% (95% CI: 0.90–1.35%) (**Appendix Table S14**, **Figure 5A**). Marked regional variation was observed across GBD regions (**Appendix Table S14**). The highest PAFs were reported in Tropical Latin America (2.31%, 95% CI: 1.86–2.78%), Andean Latin America (2.18%, 95% CI: 1.75–2.63%), and Eastern Europe (1.96%, 95% CI: 1.57–2.36%). In contrast, East Asia (0.69%, 95% CI: 0.56–0.84%) and South Asia (0.75%, 95% CI: 0.60–0.91%) had the lowest PAFs. Despite their lower attributable fractions, South Asia and East Asia contributed substantial numbers of cases (49,443 and 25,562, respectively) because of their large populations. High-income North America contributed 26,504 attributable cases (PAF 1.53%, 95% CI: 1.23–1.85%), whereas Australasia had the highest PAF among high-income regions (1.46%, 95% CI: 1.17–1.76%) but a relatively small absolute burden (890 cases). Detailed estimates for all 204 countries and territories are provided in **Appendix Table S14**.

**Figure 5:**
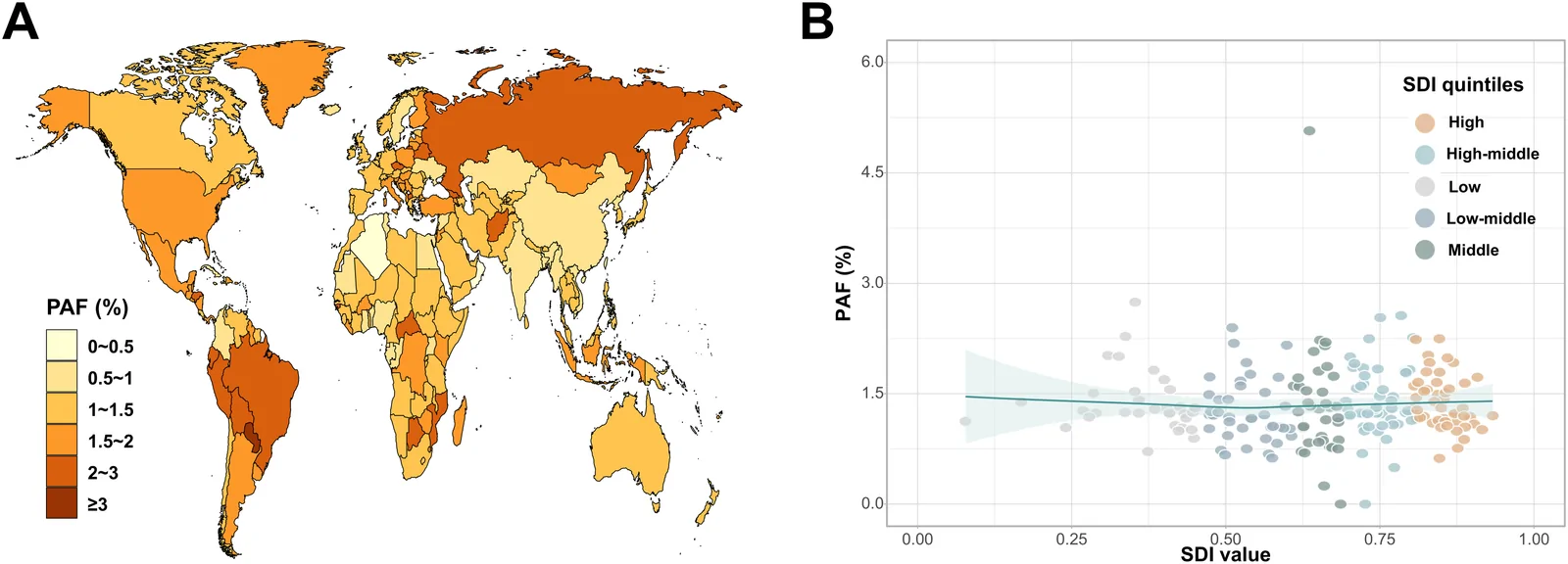
Population attributable fraction of incident type 2 diabetes attributable to COVID-19 infection in 2023 and its association with the Socio-demographic Index. **(A)** Global choropleth map depicting the estimated PAF (%) of incident T2D attributable to COVID-19 infection in 2023 across 204 countries and territories. **(B)** Scatter plot illustrating the association between country-level PAF (%) and Socio-demographic Index (SDI) value in 2023. Each data point represents one country or territory (n = 204). The fitted line represents a linear regression of PAF on SDI. Shaded band indicates the 95% confidence interval of the regression estimate.

India (33,283 cases), the United States (25,582), China (24,373), Brazil (14,408), and Mexico (14,282) bore the largest numbers of COVID-19-attributable T2D cases, whereas Paraguay (5.07%), Guinea (2.74%), Belarus (2.56%), and North Macedonia (2.53%) had the highest PAFs (**Appendix Table S15**). Across SDI strata, PAFs ranged from 0.92% in low-middle-SDI countries to 1.29% in high-SDI countries, the latter accounting for the greatest attributable burden (111,032 cases; **Appendix Table S14**). Nevertheless, country-level PAFs were not significantly associated with SDI (**Figure 5B**).

## 4 Discussion

In this comprehensive systematic review, meta-analysis, and burden-of-disease study, we found that SARS-CoV-2 infection was associated with a significantly increased risk of incident T2D, whereas no significant association was observed for incident T1D. Notably, the association with T2D was confined to studies encompassing mixed viral variants, while studies restricted to a single variant (Delta or Omicron) showed essentially no excess risk, suggesting that the observed association may be modified by viral lineage, pandemic phase, or evolving patterns of clinical management and testing over the course of the pandemic. By integrating this pooled risk estimate with contemporary Global Burden of Disease (GBD) 2023 data, we estimated that approximately 275,000 incident T2D cases worldwide in 2023—corresponding to a population attributable fraction (PAF) of 1.12%—were attributable to prior COVID-19 infection, with pronounced variation by region and level of socioeconomic development.

Our pooled estimate of a 25% relative increase in the risk of incident T2D following SARS-CoV-2 infection is broadly consistent with earlier cohort studies and meta-analyses reporting elevated diabetes risk after COVID-19 (24, 29, 44, 54, 55). Our analysis extends this literature in several ways. First, stratification by viral lineage showed that the pooled association was driven almost entirely by studies spanning mixed or pre-Omicron variants, while contemporary single-variant analyses from the Delta and Omicron era showed essentially null effects. This pattern raises the possibility that the excess risk documented earlier in the pandemic—when vaccination coverage and population immunity were lower, therapeutic options were more limited, and acute-phase management differed substantially from later practice—may not persist to the same degree with later variants and higher levels of population immunity (22, 24, 48). Second, the substantial statistical heterogeneity across studies (I² = 96.8%) warrants cautious interpretation of the pooled estimate. Although the summary RR was statistically significant (1.25, 95% CI 1.16–1.34), the 95% prediction interval (0.94–1.66) crossed the null, indicating that the magnitude of excess T2D risk may vary across populations and epidemiological contexts (56). Thus, the pooled estimate should be interpreted as an average association across the included studies rather than a uniformly applicable effect. The marked differences observed between mixed-variant and single-variant studies further suggest that viral lineage, pandemic phase, vaccination, and changes in clinical management may contribute to between-study heterogeneity. Third, by pairing the pooled risk estimate with GBD-modeled estimates of infection prevalence and diabetes incidence, we provide, to our knowledge, one of the first global-to-national quantifications of the diabetes burden attributable to COVID-19, extending the largely qualitative or single-country conclusions of prior work.

SARS-CoV-2 gains cellular entry via the angiotensin-converting enzyme 2 (ACE2) receptor, which is expressed in pancreatic islet cells, providing biological plausibility for direct viral cytopathic injury to insulin-producing β-cells (21). In addition, the profound systemic and tissue-level inflammatory response during acute infection—including cytokine release, innate immune activation, and stress signaling pathways—may further compromise β-cell function while simultaneously exacerbating peripheral insulin resistance (57). Iatrogenic mechanisms may also contribute; in particular, glucocorticoids, which are widely used in the management of moderate-to-severe COVID-19, are well established to induce hyperglycemia and may precipitate overt diabetes in individuals with underlying metabolic vulnerability (58). Beyond the acute phase, emerging evidence suggests that post-COVID-19 condition may be characterized by persistent low-grade inflammation, endothelial dysfunction, and autonomic dysregulation, any of which could contribute to sustained metabolic perturbations extending beyond clinical recovery (59). In parallel, indirect population-level effects of the pandemic should be considered. Disruptions in physical activity, dietary behavior, and routine healthcare access, alongside increased psychological stress, weight gain, and delays in pre-diabetes detection and management during lockdown periods, may have independently increased diabetes risk in both infected and non-infected populations, thereby potentially confounding observed associations (60, 61). Finally, enhanced healthcare engagement among individuals recovering from COVID-19—including increased clinical surveillance, laboratory testing, and follow-up care—may have facilitated earlier detection of previously undiagnosed dysglycemia, introducing the possibility of ascertainment bias that cannot be fully excluded in observational analyses (62, 63).

In contrast to T2D, the pooled analysis for T1D did not reach conventional statistical significance; however, the point estimate suggested a modest increase in risk (RR 1.13, 95% CI 0.99–1.30). This finding should therefore not be interpreted as evidence of a definitive absence of association. Notably, the subgroup pattern suggested that the association was more apparent in studies conducted before widespread vaccination or among unvaccinated populations, whereas it was attenuated in vaccinated populations. Vaccination may therefore represent an important contextual factor when interpreting the heterogeneous evidence on T1D following SARS-CoV-2 infection. The protective effects of COVID-19 vaccination against SARS-CoV-2 infection have been demonstrated across different populations and vaccination schedules, and increasing vaccine doses have been associated with progressively lower infection risk (16). However, the apparent attenuation of T1D risk among vaccinated populations cannot be interpreted as a causal protective effect of vaccination against post-infectious diabetes on the basis of the available study-level evidence, because vaccination status is closely correlated with calendar time, circulating viral variants, prior immunity, healthcare access, and other characteristics that may differ between study populations.

T1D is characterized by progressive autoimmune destruction of pancreatic β-cells, a process that typically evolves over months to years and is strongly influenced by genetic predisposition (e.g., HLA haplotypes) and pre-existing islet autoimmunity, rather than being triggered solely by an acute infectious exposure (64). Although viral infections have long been proposed as potential environmental accelerants of islet autoimmunity, robust human evidence specifically implicating SARS-CoV-2 in the initiation of clinically overt type 1 diabetes remains limited (65, 66). Importantly, most available studies included relatively short follow-up periods, which may be insufficient to capture incident autoimmune diabetes, given the often prolonged preclinical phase preceding clinical presentation. Accordingly, the absence of a detectable association in the current analysis should be interpreted with caution, as it may reflect limited observation time rather than true absence of risk. This is further supported by the moderate certainty of evidence and substantial between-study heterogeneity (*I²* = 83.7%), suggesting considerable uncertainty and potential differences in study design, exposure classification, and outcome ascertainment.

The absence of a statistically significant association with conventional T1D does not necessarily exclude the possibility that SARS-CoV-2 may induce transient β-cell dysfunction or an insulin-deficient phenotype that clinically resembles T1D. Several reports have described new-onset diabetic ketoacidosis or marked insulinogenic following SARS-CoV-2 infection in individuals without pre-existing diabetes, including patients who lacked pancreatic islet autoantibodies and subsequently demonstrated recovery of β-cell function and reduced or discontinued insulin requirements. These observations have led to the hypothesis that SARS-CoV-2 may precipitate a ketosis-prone or transient insulin-deficient form of diabetes distinct from classical autoimmune T1D (67–69). Although direct β-cell injury, inflammatory stress, and immune-mediated mechanisms have been proposed, the biological evidence remains heterogeneous, and the extent to which these mechanisms contribute to clinically established T1D remains uncertain (20, 70, 71). Accordingly, our null pooled association for T1D should not be interpreted as evidence that SARS-CoV-2 has no effect on pancreatic β-cell function. Rather, it may indicate that the currently available epidemiological studies, which generally rely on clinical or administrative definitions of T1D, are unable to fully distinguish persistent autoimmune T1D from transient or ketosis-prone insulin deficiency.

Whether COVID-19 vaccination can prevent or reverse this phenotype remains uncertain. Although observational evidence has suggested that vaccination before SARS-CoV-2 infection may reduce the subsequent risk of diabetes, this finding does not establish reversal of established β-cell dysfunction or insulin deficiency (72). Importantly, a recent population-based register study from Sweden found no evidence that vaccination modified the association between SARS-CoV-2 infection and incident T1D, arguing against a demonstrable protective or reversing effect of vaccination on infection-associated T1D risk (53). Therefore, current evidence does not support the conclusion that vaccination reverses SARS-CoV-2-induced insulin deficiency.

Independently of the meta-analytic findings, our GBD-based analysis reinforces that T2D remains a major and expanding global health burden (73). Substantial geographic and socioeconomic inequities persist, with low–middle SDI regions experiencing disproportionately high age-standardized incidence and DALY rates despite more constrained capacity for prevention, early detection, and long-term management. This pattern is consistent with the well-established “double burden” of communicable and non-communicable diseases observed in health systems undergoing epidemiological transition (74). Against this baseline, the addition of COVID-19-attributable T2D burden reveals a further layer of heterogeneity. Population attributable fractions were highest in Tropical and Andean Latin America and Eastern Europe, regions that experienced intense SARS-CoV-2 transmission and, particularly in the early pandemic phase, comparatively limited access to vaccination, antiviral therapies, and advanced critical care. In contrast, several highly populous countries, most notably India and China, exhibited lower PAFs but still accounted for large absolute numbers of attributable cases. This divergence underscores a critical epidemiological principle: modest per-capita risk increases can translate into substantial population-level burden when applied to large population bases (75). Diabetes is a chronic, resource-intensive condition associated with substantial morbidity, mortality, and long-term healthcare expenditure. Even a relatively small increase in incident T2D attributable to SARS-CoV-2 infection may therefore exacerbate strain on already overburdened health systems, especially in settings with high baseline cardiometabolic risk (76). These results support the integration of metabolic assessment into post-COVID care pathways, including systematic monitoring of fasting plasma glucose and glycated hemoglobin to enable early identification of dysglycemia and timely intervention (77). In addition, prevention of severe COVID-19 remains a key strategy for reducing downstream health consequences. Vaccination, early treatment, and protection of high-risk populations may not only reduce acute morbidity and mortality but may also help limit long-term metabolic complications associated with infection. Finally, our results underscore the importance of strengthening global strategies for diabetes prevention, including promotion of healthy lifestyles, early detection of metabolic disorders, and improved management of cardiometabolic risk factors.

Several limitations should nonetheless be acknowledged. First, all included studies were observational, and despite adjustment for measured covariates, residual and unmeasured confounding—including differences in body mass index, socioeconomic status, and pre-pandemic metabolic risk—cannot be excluded. Second, ascertainment of both COVID-19 exposure and incident diabetes varied across studies (e.g., PCR-confirmed versus diagnostic-code-based case definitions, and differing minimum follow-up windows), which may have introduced misclassification and detection bias, particularly given that individuals with confirmed COVID-19 likely had greater healthcare contact, and thus greater opportunity for incidental diabetes diagnosis, than uninfected comparators. In addition, pre-diabetes status before SARS-CoV-2 infection could not be determined because pre-exposure glycemic testing was generally not reported in the include studies. The 28-day minimum follow-up reduced misclassification from transient acute hyperglycemia but did not address pre-existing pre-diabetes. Some participants with pre-existing pre-diabetes may therefore have been classified as incident diabetes cases, potentially contributing to residual confounding and detection bias. Third, most included cohorts were conducted in high-income settings, and region-specific estimates of the association between SARS-CoV-2 infection and incident T2D were unavailable. We therefore applied a common pooled relative risk across populations, which may not adequately reflect geographic differences in susceptibility, baseline metabolic risk, healthcare access, vaccination and treatment patterns, viral exposure, or diabetes ascertainment. Consequently, the country- and region-specific PAFs should be interpreted as standardized model-based estimates rather than direct estimates of geographically varying causal effects. This limitation may introduce geographic bias and affect the precision of attributable burden estimates, particularly in low- and middle-income settings. Because region-specific relative risks were unavailable, we could not account for such geographic variation in the PAF estimates. Future studies from underrepresented low- and middle-income populations are needed to improve the geographic validity of these estimates. Fourth, our PAF and attributable-case calculations rely on modeled GBD estimates of COVID-19 prevalence and diabetes incidence, which themselves carry inherent uncertainty and are subject to the modeling assumptions of the GBD framework; future updates to these inputs could alter absolute attributable case counts even if relative regional patterns remain stable.

## 5 Conclusion

In summary, SARS-CoV-2 infection was associated with a modest increase in pooled incident type 2 diabetes risk, predominantly in studies spanning the pre-Omicron period; however, substantial between-study heterogeneity and a 95% prediction interval crossing the null indicate that this association may not be consistent across populations and settings. Global extrapolation of these estimates suggests that COVID-19 infection may have contributed to more than 250,000 incident cases of type 2 diabetes in 2023, with a disproportionate relative burden in Latin America and Eastern Europe and the largest absolute burden in highly populous countries. Collectively, these findings indicate a measurable but temporally constrained contribution of SARS-CoV-2 to global type 2 diabetes incidence. They support the incorporation of post-COVID metabolic monitoring into routine clinical practice and prevention strategies, and underscore the need for further mechanistic and longitudinal studies to clarify causality and long-term risk trajectories.

## Data Availability

The original contributions presented in the study are included in the article/**Supplementary Material**. Further inquiries can be directed to the corresponding authors.
