# Supplementary material for: Global burden of type 2 diabetes attributable to SARS-CoV-2 infection
Source: Front Immunol. 2026 Oct 16;17:1969341. doi: 10.3389/fimmu.2026.1969341 (PMC13719592; doi:10.3389/fimmu.2026.1969341)
Supplement: Supplementary file 1 [file SupplementaryFile1.docx]

**Figure S1.** Sensitive analysis on the association of SARS-CoV-2 infection with type 2 diabetes in mixed-variant real-world subgroup

**Figure S2**. Association of SARS-CoV-2 infection with type 2 diabetes in mixed-variant real-world subgroup after excluding the extreme unvaccinated cohort (Taylor), 2022 to 2026

**Figure S3**. Begg’s funnel plot for publication bias of type 2 diabetes in the mixed-variant subgroup

**Figure S4**. Sensitive analysis on the association of SARS-CoV-2 infection with type 1 diabetes

**Figure S5.** Association of SARS-CoV-2 infection with type 1 diabetes after excluding two extreme studies (Ward et al. and Taylor unvaccinated cohort)

**Figure S6.** Begg’s funnel plot for publication bias of type 1 diabetes
